# 2481 A collaborative neurology-emergency medicine rapid outpatient clinic for the management of TIA and minor stroke in the emergency department

**DOI:** 10.1017/cts.2018.153

**Published:** 2018-11-21

**Authors:** Bernard P. Chang, Rachel Mehendale, Eliza Miller, Benjamin Kummer, Joshua Willey, Mitchell Elkind

**Affiliations:** 1 Columbia University Medical Center; 2 Irving Institute for Clinical and Translational Science

## Abstract

OBJECTIVES/SPECIFIC AIMS: Current practice frequently dictates hospitalization for TIA and minor stroke (TIAMS) in order to obtain comprehensive evaluation of stroke risk factors and mechanism. Inpatient hospitalization is often done to expedite workup and to coordinate care although may be associated with nosocomial risks and increased healthcare cost. However, a subset of these patients who do not have debilitating deficits may not require inpatient hospitalization. We conducted a pilot study to assess the feasibility of conducting rapid outpatient stroke evaluations in low risk patients with TIAMS without disabling deficits. METHODS/STUDY POPULATION: The rapid access clinic was initiated at a single-site urban tertiary care facility for outpatient evaluation of TIAMS within 24 hours of emergency department (ED) evaluation. Patients were selected using a decision tool identifying presumed low-risk TIAMS seen in the ED. Criteria included medical (e.g., no disabling deficit, no thrombolytic agent given, negative CT for hemorrhagic stroke) as well as social criteria (e.g., patient ability to follow-up as an outpatient). We evaluated rates of noncompliance with post-ED follow-up, need for hospitalization from clinic, and 90 day stroke and health outcome data. RESULTS/ANTICIPATED RESULTS: Between December 2016 and December 2017 a total of 93 TIAMS patients seen in the ED were recommended for the rapid access clinic utilizing the decision tool. Of these patients, 94.5% (86) were evaluated within 24 hours of ED discharge. Only 2 patients (2.4%) who received outpatient evaluation required hospitalization; 61 (71.8%) patients had TIAMS on final evaluation in clinic. DISCUSSION/SIGNIFICANCE OF IMPACT: Our pilot data suggests that for a subset of patients, rapid outpatient evaluation may be a feasible and safe strategy for TIAMS management. Future work exploring such strategies may help improve TIAMS outcomes and reduce ED crowding and unnecessary hospital admissions.

